# Defining and measuring gender: A social determinant of health whose time has come

**DOI:** 10.1186/1475-9276-4-11

**Published:** 2005-07-13

**Authors:** Susan P Phillips

**Affiliations:** 1 Queen's University Department of Family Medicine 220 Bagot St, Kingston, Ontario K7L 5E9 Canada

**Keywords:** gender, women's health, social determinants, longevity

## Abstract

This paper contributes to a nascent scholarly discussion of sex and gender as determinants of health. Health is a composite of biological makeup and socioeconomic circumstances. Differences in health and illness patterns of men and women are attributable both to sex, or biology, and to gender, that is, social factors such as powerlessness, access to resources, and constrained roles. Using examples such as the greater life expectancy of women in most of the world, despite their relative social disadvantage, and the disproportionate risk of myocardial infarction amongst men, but death from MI amongst women, the independent and combined associations of sex and gender on health are explored. A model for incorporating gender into epidemiologic analyses is proposed.

## Background

A growing literature on the social determinants of health, suggests explanations for many population and individual level health outcomes are not attributable to biology. Income, income inequality, social connectedness, and social capital all show some association with health and illness [1-6]. This paper explores the meaning of gender as another of these social determinants of health, and proposes an epidemiological framework for including gender as an independent variable in future research.

## Sex versus Gender

The usefulness of distinguishing between sex and gender, a common practice in the social sciences, has begun to percolate into the language of prevention, etiology and causation within health care. Sociologists describe sex as the relatively unchanging biology of being male or female, while gender refers to the roles and expectations attributed to men and women in a given society, roles which change over time, place, and life stage. Genetic makeup and hormone profile are both examples of sex, that is, of biologic characteristics, which tend to be constant across societies. Gender is a social, rather than a biological construct, and varies with the roles, norms and values of a given society or era. Being able to bear a child is, fundamentally, a function of biology, while expectations about the imperative to bear children, the nature of parenting, or the status associated with being a mother are more closely linked to gender roles and expectations.

Gender has an impact on health in a variety of ways. Powerlessness and lack of control underlie much of the exposure to HIV/AIDS amongst women in Africa. Disproportionate barriers (that is, relative to men) in access to resources such as food, education, and medical care, disadvantage women throughout the developing world. Risk taking behavior is the norm amongst males throughout the world. Socially defined traits often stereotype men and women as having fixed and opposite characteristics such as active (male)/ passive (female), rational (male)/ emotional (female) [7,8]. The language of medicine and its underlying philosophy have, and may still equate male with normal, leaving female to be considered as "other" or, perhaps, abnormal [9]. Both women's and men's occupational and behavioural roles, constrained by social norms, can result in hazardous, though different exposures to dangers and illness [10]. Any of these aspects of gender may intercede in the pathway from an individual to his or her health.

## Differentiating Gender and Women's Health

While the content of women's health is quite clearly restricted to women, both men and women are subject to the health effects of gender. Perhaps because the deleterious impact of gender appears to burden women disproportionately, the literature on gender as a determinant of health that has begun to appear is generally about women. At a population level, in most countries of the world, women have more limited access to, and less control over, resources, and over their bodies and lives, than do men. Women appear to suffer more negative health consequences of inequalities between the sexes. The very recent increase in research on sex and gender differences, with it's focus on women, has begun to correct a history of generalization from the male subject to the female population [11-13]. Government funding agencies have given priority to research in women's health in recent decades to correct these past imbalances in research. Although efforts to report sex differences in studies and clinical presentations are not always optimal, they are well underway. There remains, never the less, much work to be done in delineating the associations between gender and health, particularly the health of men.

The philosophy of the diverse and extensive literature on women's health published over the preceding twenty years diverges into four groups over the question of gender. The first group does not question biology as the sole determinant of health but calls for an end to the discrimination that made the biology of women invisible. For these authors optimizing women's health requires attention to the diseases that are unique to, more common amongst, or present differently in women but accepts, as a biologic imperative, the male, female differential in incidence of these diseases. The limitations of such an approach are two-fold. Firstly, women continue to be defined in terms of men. Secondly, biology defines being, while social determinants vanish. A second group introduces the term gender but uses it interchangeably with sex [14-17]. The third group links the health of women as patients to the well being of women as health care providers in a traditionally patriarchal health care delivery system. Much influenced by "the glass ceiling" literature describing the challenges women face in advancing to leadership positions, this group suggests that only after models of care change will health outcomes for women improve. Of most relevance to this paper is the fourth group, which defines gender as a social determinant of the health of both men and women, but grapples with how sex and gender interact, and with the specifics of how gender shapes individual health [14,18]. The remainder of this paper is concerned with these questions.

## Interactions Between Sex and Gender

Neither sex nor gender can adequately be examined as variables associated with health but independent of each other. Gender interacts with biology, in every society, although the results of that interaction vary from setting to setting.

Sex differences in life expectancy offer one of many examples of the interaction between sex and gender in health outcomes. In most of the world women outlive men, the exceptions being the least developed countries, where extremely high birth rates and significant human rights inequalities disproportionately disadvantage women. Despite having had most of the social determinants of health in their favor, men have higher mortality rates for all 15 leading causes of death, and a life expectancy about seven years shorter than women's in the United Kingdom, as in most Western countries [19]. Mortality data from 1995 for Canada's largest province, Ontario, show the same contradiction [20]. Government documents, however, report the numbers but do not explore the striking sex differences in longevity. Sub-analysis of the data explain the interconnectedness of sex and gender and shed light on the apparent contradiction inherent in both the UK and Canadian data. Between birth and age 45, there are 1,812 male deaths, of which 1,372 (76%) are due to motor vehicle accidents, suicide, and AIDS, leaving 440 deaths unrelated to behavior. Although the male excess of deaths from car accidents may, in part be attributable to greater distances driven and not behavior while driving, the male "relationship" with the automobile is almost certainly another aspect of gender roles. Only 308 (33%) of the 936 female deaths are explained by such behavior. When non-risk taking causes of death are isolated from the data, women under age 45 have a mortality which is 1.43 times that of men's. Over age 45 the leading causes of death for both men and women are chronic diseases. Men die of heart disease in equal numbers but at a younger age than do women. With increasing age the number of deaths for women creeps upward to equal that of men. Male gender roles as manifest by risky behavior around drinking, driving, and sex, account for virtually all excess male mortality below age 45, and approximately 50% of the excess below age 60. This data suggest that gender is a central, although not the sole cause of the shorter life expectancy of men in western society. How much of this gendered behavior actually arises from a male, historic, biological imperative to dominate other males and thereby win a mate to procreate is unknown. Therefore, a sex difference in longevity appears to result, in part, from a gender difference in behavior, which may, in turn, arise from the biology of sex. Defining which aspects of female advantage in life expectancy arise purely from the fixed biological attributes of sex and which are due to gender and amenable to change is challenging.

## Redefining Gender

Given the interconnectedness of the biological and the social, it might prove pragmatic to consider that gender encompasses both sex differences and the social constructs that give rise to gender differences. For example, in most developed countries myocardial infarcts are a leading cause of death for both men and women. Male risk of coronary artery disease at a younger age exceeds female risk for what appear to be biologic reasons. Because, however, the "prototype" of the heart disease patient has been male, the most common presentation amongst men, that of crushing chest pain, has become the most important diagnostic clue to the presence of angina. Recent evidence demonstrates that fatigue, rather than chest pain, is, in fact, the most common symptom of angina in women [21]. The gender biases inherent in the underlying assumption that coronary artery disease is primarily a male affliction, and that those women who suffer from it will present as men may explain why women have a significantly higher post MI mortality than do their male counterparts [22]. There is no practical advantage to disentangling where sex ends and gender takes over as a cause of the sequelae of coronary artery disease. Never the less, for a physician to properly diagnose angina requires an understanding of sex differences in the symptoms associated with coronary ischemia, and a rejection of the gender stereotype that men, and not women, are the bearers of this illness. By defining gender as the composite of both social and biological health effects associated with being either male or female, researchers may more easily move on to studying those effects, without getting stuck at enumerating sex differences.

## Measuring gender effects: epidemiology challenges

Defining gender is a necessary prerequisite but not a sufficient solution to the problem of developing a methodology of its measurement. The impact of gender as a social determinant of health is likely a composite of the effects of relative power, autonomy, poverty, and marginalization, within, and across, societies and cultures. As gender is, by definition, a social or population level determinant of health, its consequences at an individual level are less tangible. There are no randomized controlled clinical trials (RCTs), the "gold standard" of individual level research, that measure the health effects of gender. Gender defies "packaging" as an etiologic agent of disease nor could it appear in a list of differential diagnoses for a set of clinical findings.

How should the associations between gender and illness be studied? The process could involve identifying a cohort from numerous countries, prospectively tracking health outcomes such as longevity, mental health, or incidence of a variety of morbidities, and identifying which non-biological inputs (the usual ones being age, sex, education, income, lifestyle risk factors like smoking, alcohol and drug use) are associated with adverse outcomes. Using regression analysis, the effect of socioeconomic factors on health could be identified and isolated from other inputs, rather than controlled for and eliminated (as happens in an RCT where the randomization equalizes the effect of these social phenomena between the study and control group, but precludes analysis of the association of these phenomena with the outcomes in question since they are effectively deleted prior to analysis). The challenge of how to insert gender as an independent variable, that is, into the left side of such a regression analysis, would, however, still remain.

The interconnection between gender and socio-economic status necessitates addressing both in analyzing associations with health and illness. In thinking about this, the epidemiologic constructs of 'within group' and 'between group' variation may be useful. The concept of gender could include differences in socio-economic and cultural determinants of health between men and women. If the groups being studied or compared are men and women, the between group variations would then be summed up by gender. There remain, however, within group variations because not all women are the same. Data examining the percentage of births attended by trained personnel and aggregated by the level of the mother's education consistently favour the more educated and demonstrate variation in access to care [23]. Within the grouping 'women' social determinants such as education or income often account for differences in power or access to care, and, ultimately, to health.

## A gender coefficient

Specific coefficients, or composites of several variables have been developed to measure some of the social determinants of health. Levels of income or education can be categorized. Income inequality, another of the well-studied social determinants of health can be quantified in several ways. Human rights, although more vague, can be measured via proxy variables such as participation of women in government and public life. As yet, no one has proposed a proxy measure for gender. Indicators of human rights may approximate gender when the health of women is the outcome being examined. Similarly income, income distribution, and access to education or health care are likely colinear with gender in the measure of women's health. Even more important may be the interaction between these variables in answering questions such as how the health of women in a relatively wealthy, but repressive country compares to women's well being in a less wealthy, but more egalitarian country. Multilevel analyses may capture individual variations in wealth or freedom within population level research. More problematic is identifying proxies for gender when examining men's health. Perhaps variables that measure acceptance of violence in a society (e.g. prevalence of gun ownership) touch on gender as a determinant of the health of men. Unfortunately there is, at present, no gender equivalent of the Gini co-efficient, the summative measure of income inequality in a community.

## Summary

Within the past two decades, in response to the historic under- or mis-representation of women in the research that shapes medical practice, major funders such as the NIH in the US, developed more inclusive guidelines for research methodology and funding. Concurrent with this, and likely as a result of it, a literature on women's health began to appear. Subsequently, the construct of gender was borrowed from the social sciences to broaden etiologic concepts in the area of women's health beyond biologic differences between the sexes. Although authors often use the terms women's health and gender interchangeably, gender has a wider scope, which facilitates discussion of the effects of social norms and expectations on the health of both women and men. Perhaps, because to date, no gender index or composite of variables that, together, would be a measure of gender, has been proposed, there are few, if any studies of the associations between gender and health. In the context of health outcomes, defining gender to include both biologic and social aspects of being male or female, and considering which measurable variables could form a gender co-efficient should enable research to move forward. Using some of the proposals from this paper, indices of gender may, in the future, become part of analyses of how social factors impact on health.
